# The Impact of Diet on Long-Term Oncological Outcomes: Investigating Nutritional Mechanisms in Cancer Prevention Management and Prognosis

**DOI:** 10.3390/nu18060881

**Published:** 2026-03-10

**Authors:** Shubana Hayat, Junaid Ahmad, Sara Naeem, Faiza Yaseen, Sania Aamir, Francesca Guida, Livio Luongo, Sabatino Maione

**Affiliations:** 1 Department of Experimental Medicine, Università Degli Studi Della Campania “L. Vanvitelli”, 80138 Naples, Italy; shubana.hayat@unicampania.it (S.H.); sara.naeem@unicampania.it (S.N.); faiza.yaseen@unicampania.it (F.Y.); sania.aamir@unicampania.it (S.A.); franc.guida@gmail.com (F.G.); sabatino.maione@unicampania.it (S.M.); 2 Department of Experimental Medicine and Surgery, University of Rome Tor Vergata, Via Montpellier 1, 00133 Rome, Italy; ofridai116@gmail.com

**Keywords:** long-term oncology, nutrition, metabolism, inflammation, gut microbiota and epigenetics

## Abstract

Long-term oncological outcomes are significantly influenced by dietary patterns and nutritional status. Emerging evidence suggests that specific nutrients and dietary behaviors modulate the biological pathways involved in cancer initiation, progression, and therapeutic response. Understanding these nutritional mechanisms is essential for optimizing cancer prevention strategies, improving treatment efficacy, and enhancing long-term prognosis. Dieting is a modifiable factor influencing cancer prevention, progression, and survivorship. This review is a molecular, clinical, and epidemiological data amalgamation that aims to figure out the first of the three aspects, i.e., how dietary patterns and nutrients affect carcinogenesis, therapeutic tolerance, and long-term outcomes in long-term oncology. The current review moves from diet-dependent core cancer mechanisms that lead cancer pathways through metabolic reprogramming, inflammation, oxidative stress regulation, and epigenetic alterations. Protective dietary patterns, e.g., plant-based, Mediterranean-style, fiber-rich, and omega-3-fed diets, typically provide lower oxidative and inflammatory loads while also facilitating immune surveillance and metabolic stability. Therapy-personalized nutrition that is high in energy–protein and functional foods is instrumental to treatment tolerance, reduction in complication incidence, and cachexia relief. The newest research highlights the significant influence of epigenetic remodeling and the gut–brain–immune axis as the main processes that connect nutrition to tumor behavior and psychosocial outcomes. Translation into clinical practice changes is still dependent on thoughtfully designed trials, the existence of standard guidelines, and the provision of equal access to digital nutrition tools, despite this advancement. Diet is positioned as a low-toxicity co-therapeutic strategy that supports prevention, treatment efficacy, and long-term survivorship.

## 1. Introduction

Long-term oncology refers to cancers that experience long disease courses, with relapse or metastasis, and which require long-term commitment to management rather than being outrightly cured [1,2]. It sees cancer not simply as an isolated event but as a systemic condition that needs continuous treatment, metabolic, and survivorship care. Compared to acute cancer, prolonged cancers require medical treatment together with various lifestyle changes such as proper nutrition, physical activities, and psychological support [3]. During the last few years, the comprehension of cancer biology has gone far beyond the scope of genetics and pharmacotherapy to incorporate lifestyle factors, of which diet is the most influential [4]. Food is no longer seen just as a need to be fulfilled; it is considered a major biological factor that can change cellular metabolism, oxidative balance, immune modulation, and even gene expression [5,6]. Scientific evidence has been very clear over time that what we eat has a huge impact on the starting and the growth of cancer cells. This, thus, makes nutrition a very important component in integrative oncology [7,8]. Increased research is coming out that points to the significance of dietary habits in determining cancer results. Diets loaded with fruits, vegetables, whole grains, and good fats like those found in Mediterranean or plant-based diets have been proven to make one less likely to get these cancers that develop over time, among which are breast, colorectal, and prostate cancer [9,10]. On the other hand, an elevated intake of processed meats, refined carbohydrates, and saturated fats has been linked to the development of cancer through various pathways including insulin resistance, chronic inflammation, and changes in gut microbiota [11,12]. Hence, altering one’s diet is a potentially attractive and feasible way to lower the incidence of cancer and to raise the quality of life of patients not only during the therapy period but also in the phase of survivorship [13,14]. The worldwide cancer burden keeps getting worse everywhere [14]. The World Health Organization (WHO) estimates that cancer is the cause of nearly 10 million deaths globally every year and forecasts are indicating a massive increment of the cases over the following decades [15,16,17]. Based on epidemiological analyses from the World Health Organization, the Global Burden of Disease project, and the World Cancer Research Fund, around one third to one half of cancers result from lifestyle factors that can be changed, such as diet, obesity, physical inactivity, and alcohol consumption [18,19]. The fraction varies depending on the region and cancer subtype, with the greatest variations for colorectal, breast, liver, and endometrial cancers [20,21]. Such figures highlight the major preventive capacity of scientifically dietary and lifestyle-based interventions to be integrated in cancer control strategies worldwide [22]. Excessive nutrition, obesity, and diet-related metabolic disorders have been identified as leading causes that can not only bring about the initial occurrence of cancer but also its relapse [23]. The worldwide tendency to consume high-calorie, low-fiber, and nutrient-deficient foods has led to the impairment of the body’s natural defense mechanisms against oxidative stress and immune dysregulation, which are phenomena at the core of carcinogenesis [24]. Moreover, the differences in eating habits between the regions point to a direct relationship between nutrition and cancer rates; as an example, Western societies with more red and processed meat consumption show higher colorectal cancer incidence, while Asian diets abundant in plant-based foods and polyphenols correspond to a lower risk of cancer [25,26]. These discrepancies emphasize the importance of culturally appropriate dietary interventions in cancer prevention and care worldwide [27]. Although there is a vast amount of research on diet and cancer, the link that connects nutrition and oncology is still complex and not entirely comprehended [28]. A large number of studies concentrate on the effects of isolating one dietary ingredient or one cancer type and often overlook the continuous progression of the disease, the response to treatment, and the phase of survivorship [29]. Hence, this review’s intention is to synthesize the existing evidence across the cancer trajectory to shed light on the influence of diet on molecular pathways, therapeutic efficacy, and long-term prognosis [24]. The primary goal is to chart the mechanisms through which food nutrients influence cancer development. After that, the focus is on the role of nutrition in treatment tolerance, and finally, an assessment of post-treatment dietary habits in relation to relapse and survival is made. This paper serves as a bridge between the molecular, clinical, and epidemiological perspectives to arrive at a single conclusion. Essentially, diet will become an important instrument in cancer management for both prevention and therapy. The goal of dietary oncology research is to make personalized nutrition plans possible that go hand in hand with precision medicine, thereby enhancing the healthcare quality and survival rate of cancer patients globally. Throughout this review, the authors make a clear distinction across the board between mechanistic findings coming from preclinical models and associative evidence from observational studies on the one hand, and the results of randomized clinical trials on the other hand. Considering the complexity of cancer biology and interindividual variability, most diet–cancer relationships ought to be seen as associations or dietary modulations with a possible influence, rather than direct causal effects. As far as the supporting evidence is concerned, the level of evidence is specified so that a balanced and evidence-based interpretation can be made.

## 2. Diet and Cancer Prevention

Diet is a major factor that affects cancer risk through its impact on inflammation, oxidative stress, metabolism, and immune function [29]. Moving away from mostly plant-based traditional diets towards processed, energy-dense foods is at the root of the increasing cancer cases worldwide, especially in developing countries where the lifestyle is rapidly changing [30]. Many population-based studies indicate that about 33% of cancers could potentially be prevented through diet and lifestyle choices. The quality of food, its nutrient content, and macronutrient composition have a significant influence on the biological mechanisms that determine whether cells will remain healthy or become cancerous [31,32]. Plant-based diets that mainly consist of fruits, vegetables, whole grains, legumes, and nuts have been found to significantly reduce the risk of cancer [33]. These kinds of foods are full of antioxidants and phytochemicals such as flavonoids, carotenoids, and polyphenols which absorb the reactive oxygen species and protect DNA from damage [34]. For instance, sulforaphane in cruciferous vegetables and lycopene in tomatoes help activate endogenous detoxification enzymes while simultaneously modulating inflammatory signaling pathways [35]. Fiber in the diet improves the diversity of the gut microbiota and thus it can generate short-chain fatty acids, for example, butyrate, which are beneficial to the colon and the immune system [36]. People following Mediterranean-style diets that mainly consist of olive oil, fish, and plant foods have lower chances of getting colorectal, breast, and prostate cancers as a result of the anti-inflammatory and antioxidant effects of these diets [10,37].

Conversely, the excessive intake of saturated fats, trans fats, and refined sugars causes metabolic disturbances that lead to cancer development [38]. Diets rich in saturated fats have been shown to be correlated with elevated inflammatory cytokine production and oxidative stress. In addition, trans fatty acids have been found to cause insulin resistance [39]. On the other hand, long-chain omega-3 polyunsaturated fatty acids (PUFAs), like EPA and DHA, possess anti-inflammatory effects and probably regulate eicosanoid signaling pathways [40]. Accumulating epidemiological and translational evidence indicates that long-chain *n*, 3 polyunsaturated fatty acids (PUFAs), mostly eicosatetraenoic acid (EPA) and docosahexaenoic acid (DHA), can modulate cancer progression through inflammatory signaling regulation, changes in membrane lipid composition, and immune cell function [41]. Studies that observe breast and colorectal cancer patients have found associations between plant and animal marine-derived *n*, 3 PUFA consumption and reduced risk of recurrence or better survival outcomes, although effect sizes differ among cohorts [42]. At the molecular level, EPA and DHA displace the production of arachidonic acid-derived eicosanoids resulting in an increase in specialized pro-resolving mediators that dampen chronic inflammation in the tumor microenvironment [43]. *n* In addition, *n*, 3 PUFAs can affect the fluidity of the membrane, the clustering of the receptors, and the cellular signaling pathways that eventually control proliferation and apoptosis [44]. Although only a few randomized clinical trials with definite survival endpoints have been conducted, current data backs up the role of *n*, 3 PUFA-rich dietary patterns as adjunctive therapies in long-term cancer management [45]. Epidemiological data on *n*, 3 PUFA consumption and cancer progression, however, still yield mixed results despite the support of such research. The variation in the results of these studies may be due to the differences in the dose, duration of intake, initial dietary patterns, and the ratio of *n*, 6 to *n*, 3 fatty acids, among other things [46]. Furthermore, there is a huge difference in how different individuals convert plant-based alpha-linolenic acid (ALA) into EPA and DHA, which adds to the metabolic variation [47]. Besides the limitations of the study design such as the use of food frequency questionnaires, residual confounding, and reverse causality, these may also be reasons for the inconsistent findings. Hence, the existing data can be regarded more as an association than a direct causation, thus emphasizing the importance of well-designed, stratified, prospective randomized controlled trials [48].

Diets rich in sugars and high-glycemic foods result in elevated insulin and insulin-like growth factor-1 levels, which promote abnormal cell proliferation and inhibit apoptosis; thus, these cancers originate mainly in the colon and pancreas. Substituting refined carbohydrate products with complex and fiber-rich ones is beneficial for glucose metabolism regulation and reduction in systemic inflammation [49,50]. Furthermore, cancer preventive measures may be enhanced with the help of micronutrients like vitamins A, C, D, and E, and trace elements such as selenium and zinc that are involved in maintaining genomic stability and immune competence [32]. Polyphenols and other dietary components, e.g., curcumin, resveratrol, and green tea polyphenols, are involved in the modulation of signaling pathways affected in inflammation, cell growth, and apoptosis [51]. Moreover, a well-functioning gut microbiome, which is nourished by fiber and probiotic foods, is capable of strengthening intestinal barriers and detoxification mechanisms [52] (Table 1).

While overall diet patterns like plant-based and Mediterranean diets continuously show an association with lower cancer risk and better recovery outcomes, a growing number of studies indicate that the impact of nutrition might vary depending on the molecular subtype and metabolic phenotype of the tumor [66]. Biological heterogeneity in chronic tumors is so great that it involves differences in how hormone receptors are expressed, genomic instability, reliance on glycolysis, changes in lipid metabolism, and the composition of the microbiome, all of which can affect how the tumor responds to dietary interventions [67]. In breast cancer, estrogen receptor-positive (ER+) and progesterone receptor-positive (PR+) tumors are often sensitive to insulin and insulin-like growth factor, 1 (IGF-1) signaling pathways, which activate PI3K/Akt/mTOR cascades associated with proliferation and endocrine resistance [68,69]. Thus, dietary strategies, which improve glycemic control, such as Mediterranean or low-glycemic dietary patterns rich in fiber, whole grains, and unsaturated fats, theoretically reduce insulin-driven tumor-promoting signals in hormone-sensitive disease [70]. On the other hand, triple-negative breast cancer (TNBC) is frequently marked by increased glycolytic flux and metabolic plasticity, indicating a higher glucose metabolism dependency. Therefore, this has led to the exploration of carbohydrate-restricted or ketogenic dietary approaches as potential metabolic adjuvants; however, the current clinical evidence is only preliminary and mostly limited to feasibility studies [71,72]. HER2-positive breast cancers exhibit metabolic adaptability and activation of inflammatory pathways, and omega-3 fatty acids have been studied for their ability to change eicosanoid signaling and tumor-associated inflammation. There are clinical data emerging but it is not yet conclusive [73]. In colorectal cancer, molecular subtypes such as microsatellite instability-high (MSI-high) tumors show distinct immunometabolism features compared with microsatellite-stable (MSS) disease, and the tumor-associated microbiota, especially the enrichment of *Fusobacterium nucleatum*, has been linked to carcinogenesis and progression. These results provide a basis for fiber-rich and microbiome-targeted interventions to increase the production of short-chain fatty acids, in particular butyrate, which acts as a histone deacetylase inhibitor and may facilitate the expression of tumor suppressor genes. However, the stage-specific responses and the variability between individuals need to be explored further [74,75]. In prostate cancer, especially androgen-sensitive disease and patients on androgen deprivation therapy (ADT), metabolic disturbances like insulin resistance and increased adiposity are frequent and may lead to inflammatory signaling. Therefore, Mediterranean-style dietary patterns and weight management strategies may reduce systemic metabolic stress and enhance long-term outcomes [76,77]. In addition, omega-3 fatty acids have shown biological activity in altering inflammatory microenvironments in prostate tumors, and in vitro clinical trials were suggestive of possible effects on proliferation indices [78]. Glioblastoma is a highly glycolytic cancer with a huge dependency on glucose metabolism, which is why ketogenic diets have been considered as supplementary strategies for metabolic restriction; although early-phase studies show that the approach is feasible and safe, there is still very little evidence of its effectiveness [79]. Altogether, these findings highlight the necessity of incorporating tumor molecular and metabolic profiling into nutritional oncology paradigms, thus moving towards a precision nutrition approach in long-term cancer treatment (Table 2).

## 3. Nutritional Mechanisms in Cancer Progression

**Figure 1:**
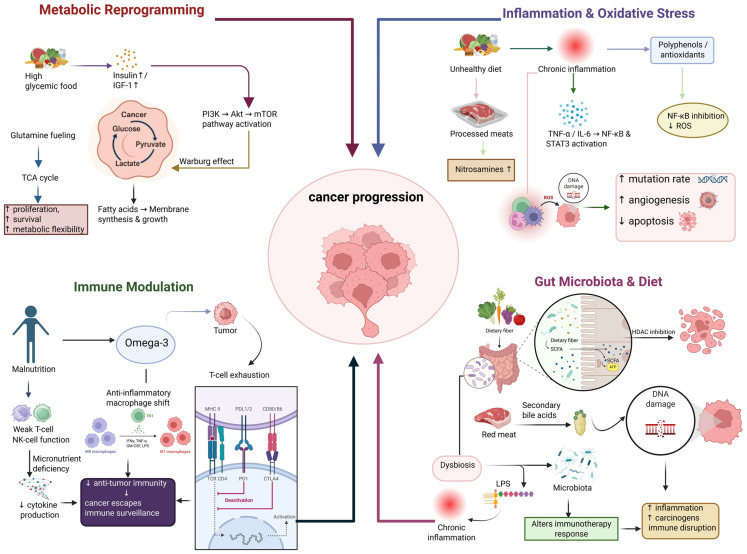
An integrated model showing the role of food in the modulation of cancer progression through metabolic reprogramming, inflammation and oxidative stress, immune modulation, and gut–microbiota interactions. Dietary habits affect the development of neoplasms due to changes in molecular signaling pathways, immune responses, and the tumor microenvironment. Colored sections represent the major biological pathways (metabolic reprogramming, inflammation and oxidative stress, immune modulation, and gut microbiota interactions), while arrows indicate directional relationships and regulatory effects leading to cancer progression.

Nutrition has become one of the major factors in the environment that has a significant impact on the origin, development, and treatment response of cancer [29]. The relationship between diet and cancer biology is a highly complex one and involves various factors such as metabolic changes, chronic inflammation, oxidative stress, hormonal regulation, and immune modulation [89]. To support their rapid growth, cancer cells manipulate nutritional and metabolic pathways; they also eliminate apoptotic mechanisms and spread to other organs (Figure 1). In addition to that, dietary habits influence gut microbiota, which ultimately determines immune function, inflammation, and even drug metabolism [90]. An adequately balanced diet enriched with bioactive compounds such as polyphenols, flavonoids, omega-3 fatty acids, and antioxidants has been proven to be protective by altering molecular signaling pathways, reducing oxidative DNA damage, and facilitating immune surveillance [91]. However, overconsumption of red and processed meat, refined sugars, alcohol, and trans fats can result in the acceleration of oncogenesis to insulin resistance, increase growth factor levels, and induce chronic inflammation. Therefore, the nutritional milieu not only influences the risk of cancer but also determines cancer aggressiveness and the patient’s response to treatment [23].

### 3.1. Metabolic Reprogramming and Dietary Factors

Cancer cells can act a deep metabolic reprogramming to allow them to multiply indefinitely and to stay alive [66]. One of the major features of this reprogramming is the Warburg effect which is the preference of aerobic glycolysis for energy production, even when enough oxygen is present, followed by the production of lactate [92]. This allows them to have biosynthetic intermediates for nucleotide, lipid, and protein synthesis, which are required for tumor growth [93]. The way we eat has a major influence on these metabolic processes. High glycemic index foods lead to a sustained insulin and insulin-like growth factor-1 (IGF-1) signaling that causes the oncogenic pathways such as PI3K/Akt/mTOR and RAS/MAPK to be activated, which increase cell proliferation and survival [94]. On the other hand, a low-carbohydrate or ketogenic diet may limit glucose availability and lower insulin levels, thereby inhibiting tumor growth in cancers like glioblastoma and breast cancer [95]. Moreover, amino acid metabolism is equally important. Cancer cells often change their metabolism in such a way that they become dependent on certain amino acids, e.g., glutamine and methionine. A diet low in these amino acids can, in theory, be a diet that deprives cancer cells of the necessary substrates for biosynthesis and redox balance [96]. Just like that, fatty acid metabolism matters a lot as well-saturated fats and omega-6 fatty acids promote inflammation and cancer development while omega-3 fatty acids (EPA, DHA) prevent the formation of inflammatory eicosanoids and assist cancer cells in apoptosis [97]. In addition to that, micronutrients such as vitamin D, selenium, and zinc have an impact on metabolic signaling and mitochondrial function [98]. Vitamin D influences the regulation of the cell cycle and differentiation through the vitamin D receptor (VDR) pathway, whereas selenium compounds improve the activities of antioxidant enzymes that eliminate ROS-induced mutations [99].

Still, a significant part of the evidence on the relationship between diet and metabolic reprogramming in cancer that we have currently comes from preclinical studies. However, clinical data are starting to show consistent translational relevance [66]. Dietary changes aimed at glycemic control can lead to a decrease in insulin and IGF-1 levels in the bloodstream of patients with breast and colorectal cancers, which may thus impact PI3K/Akt/mTOR signaling [100]. Initial-phase trials of ketogenic diets in glioblastoma and advanced solid tumors indicate that such diets are practical and metabolically effective, but survival advantages have not yet been demonstrated clearly [88]. There is good evidence that short-term fasting and protocols mimicking fasting can alleviate the toxicity of chemotherapy based on animal and small clinical studies. Restriction of methionine and other amino acid targeted strategies are at the moment being clinically tested on a small scale with early metabolic effect outcomes. However, we still require large-scale randomized controlled trials with clear oncological endpoints before formal clinical guidelines can be formulated [101].

### 3.2. Inflammation, Oxidative Stress, and Immune Modulation

Chronic inflammation along with oxidative stress are major factors that not only lead to cancer but also cancer growth. Inflammatory states that last a long time produce various cytokines such as Tumor necrosis factor alpha (TNF-α), Interleukin-6 (IL-6), and Interleukin 1 beta (IL-1β), which, in turn, activate transcription factors like Nuclear factor kappa-light-chain-enhancer of activated B cells (NF-κB) and Signal transducer and activator of transcription 3 (STAT3), thus causing these cells to proliferate in an unregulated manner, to form new blood vessels (angiogenesis), and to become resistant to programmed cell death (apoptosis) [102,103]. Furthermore, oxidants in general and reactive oxygen species (ROS) in particular, which are always characters of inflammatory processes and in some cases are released due to mitochondrial dysfunction, directly attack the DNA and consequently induce strand breaks, base modifications, and genomic instability that are the main pathways of carcinogenesis [89]. Among various factors leading to inflammation, diet plays a very significant role: high-fat, high-sugar, and processed food consumption are major causes of inflammatory signaling and oxidative load. On the other hand, diets composed of foods with antioxidative and anti-inflammatory properties will have the effect of balancing out the inflammatory processes and the oxidative load [104]. Polyphenols obtained from fruit and green tea (e.g., epigallocatechin gallate, quercetin, curcumin) are well-known agents that hinder NF-κB activation, depress COX-2 expression, and eventually lead to the apoptotic death of tumor cells [105]. In addition, vitamins C and E, carotenoids (such as β-carotene and lycopene), and minerals like manganese and selenium, can be used as cofactors for antioxidant enzymes such as superoxide dismutase (SOD) and glutathione peroxidase (GPx); thus, they can effectively scavenge ROS and maintain DNA stability [106]. Equally important diet immune modulation cannot be underestimated either. For instance, poor nutrition or micronutrient deficiency situations can cause the immune system to become weaker; thus, it will not be able to provide the same level of cytotoxic activity that T cells and natural killer (NK) cells normally do against tumor cells [107]. Nutrients which include zinc, vitamin A, and vitamin D are the main suppliers of immune cell differentiation and cytokine production processes [108]. The intake of both probiotics and prebiotics results in the activation of gut-associated lymphoid tissue (GALT) which then leads to an improvement in mucosal immunity and anti-tumor defense. Last, but not least, omega-3 fatty acids are able to lessen the production of pro-inflammatory prostaglandins and at the same time they can also contribute to the killing of tumor cells by macrophages [109].

### 3.3. Gut Microbiota and Dietary Interactions

Microbiota in the gut is a very complicated system of trillions of microorganisms, and it is one of the major factors that regulates metabolism, immunity, and cancer biology in the host system [110]. The gut fauna by itself converts food compounds into healthy metabolites that can either suppress or support cancer formation. A well-balanced diet enriched with fibers is the best friend of good bacterial species like *Lactobacillus*, *Bifidobacterium*, and *Faecalibacterium prausnitzii*, which are the main producers of short-chain fatty acids (SCFAs) such as butyrate, propionate, and acetate. One of these, butyrate, serves in colon cancer as a histone deacetylase (HDAC) inhibitor causing apoptosis and proliferation to be lowered; thus, cancer cells are inhibited in the colon [111]. The alteration of microbial composition, called dysbiosis, can lead to oncogenesis through the production of genotoxic metabolites, secondary bile acids, and lipopolysaccharides (LPS) that cause chronic inflammation and damage the epithelium [112]. Indulgence in a diet consisting of a lot of red meat and little or no fiber leads to dysbiosis, which results in an increase in carcinogenic nitrosamines and ammonia in the colon. However, plant-based diets and Mediterranean diets can not only fix the imbalance but also improve mucosal immunity and decrease the presence of pro-inflammatory bacterial species such as *Fusobacterium nucleatum*, which has been implicated in colorectal cancer progression [113]. Beyond tumor initiation and progression, the gut microbiota also plays a critical role in modulating responses to cancer therapy and treatments. Some bacterial strains, by releasing certain metabolites, stimulate the immune system, thereby enhancing the response to immune checkpoint inhibitors (like anti–PD-1/PD-L1 antibodies), while other strains could make tumors resistant to such therapies [114]. Thus, microbiome changes due to diet (prebiotics, probiotics, and symbiotic) to improve cancer treatment outcomes is the most talked about next-step approach in oncology [115].

## 4. Dietary Management During Cancer Therapy

Dietary management during cancer therapy is a very important part of the overall oncological care that should be given. It is to be noted that major interventions such as chemotherapy, radiotherapy, and immunotherapy introduce a significant physiological stress to the body [116]. Usually, these methods alter the patients’ metabolism, reduce their appetite, and injure the cells of the body that are not suffering, thereby causing patients to develop nutrient deficiencies, become fatigued, and lose muscle [117]. Nutrition in therapy repairs damaged tissues, maintains lean body mass, and thus invigorates the immune system, this way leading to better treatment tolerance and quality of life [118]. The first and foremost objective of dietetic intervention is to ensure that the nutritional status of the patient is optimal through the adequate provision of energy and protein in light of the side effects of the treatment like nausea, diarrhea, mucositis, or taste alterations [119]. Individualized nutrition plans considering factors such as cancer type, treatment phase, metabolic rate, and gastrointestinal function are the most effective for patients [120]. Consumption of balanced amounts of macronutrients and micronutrients together with sufficient hydration and the use of functional bioactive compounds can be very instrumental in lessening the side effects of the treatment, speeding up convalescence, and fortifying the body’s defenses during therapy [121] (Table 3).

### 4.1. Nutritional Support During Chemotherapy and Radiotherapy

Chemotherapy and radiotherapy are treatment methods that cause harm not only to cancer cells but also to normal cells that divide rapidly, especially in the gastrointestinal tract and bone marrow [122]. The side effects of these treatments, such as mucosal inflammation, nausea, vomiting, diarrhea, and taste changes, arise from this damage and the symptoms of these side effects severely limit food intake and nutrient absorption. Nutrition intervention during these therapies is a way of counteracting the effects mentioned above; it is a method of treatment that provides enough calories, protein, and essential micronutrients to maintain the patient’s energy and immune system [123]. Generally, the need for energy increases to about 25–35 kcal per kilogram of body weight per day, and the protein intake should be more than 1.2–1.5 g/kg/day in order to propagate cellular regeneration and repair [124]. If patients are incapable of reaching these needs through regular meals, they can be given oral nutritional supplements, and in the most severe situations, they can be fed through a tube or intravenously. Immune system and skin repair are among the main functions that require nutrients such as vitamin D, zinc, and selenium, whereas the consumption of water should be regarded as a must if the kidneys are to function normally and drug excretion is to take place smoothly [125]. In the case of radiotherapy, particularly in the areas of the head, neck, or stomach, soft, non-acidic, and protein-rich foods are advised to both cause less pain in the inflamed mucosa and facilitate mucosal healing [126]. Low levels of tissue inflammation can be induced by glutamine and omega-3-enriched supplements, while probiotics support intestinal barrier stability. The provision of nutrition support therapy during treatment not only facilitates the treatment process but also reduces the chances of infection and improves the overall health condition of the patient [127].

### 4.2. Protein–Energy Balance and Cachexia Prevention

Cancer cachexia is a metabolic syndrome of the severest kind characterized by the progressive loss of body weight, muscle atrophy, and systemic inflammation, and is usually very resistant to typical nutritional interventions [128]. This condition comes about due to the tumor-induced changes in protein and energy metabolism and includes increased energy expenditure at rest and impaired anabolic signaling. Therefore, it is a must to maintain protein–energy balance throughout treatment so as to be able to maintain muscle mass and physical function [129]. Typically, one is advised to take protein not less than 1.5 and not more than 2.0 g/kg/day and it is recommended to focus on quality proteins such as eggs, fish, poultry, dairy, soy, and legumes [130]. Energy requirements are most of the time between 30 and 35 kcal/kg/day and thus the person is advised to combine complex carbohydrates for a steady glucose release with fats that are good for the body, like monounsaturated and omega-3 fatty acids, in order to efficiently cover caloric needs [131]. Among the omega-3 fatty acids, the one that has the most beneficial effects in terms of inflammation and anti-cachexia is Eicosapentaenoic acid (EPA) that, by inhibiting a variety of pro-inflammatory mediators such as IL-6 and TNF-α which lead to muscle degradation, brings about these effects. Besides the food measures, it is advised that a person does some mild resistance exercise and implements anti-inflammatory strategies to be able to maintain muscle function. It is still a very good thing to do nutritional screening and intervention at a very early stage so as to prevent the onset of cachexia, increase the tolerance of therapy, and extend the lifespan by maintaining body composition and energy balance [132].

**Table 3:** Summary of recommended energy–protein requirements and key nutritional components across major oncology clinical care areas.

Clinical Focus Area	Recommended Energy Intake (kcal/kg/day)	Recommended Protein Intake (g/kg/day)	Key Nutrients/Functional Components	References
Overall Nutritional Goals During Active Cancer Treatment	25–35 (adjusted to metabolic stress, inflammation, and treatment stage)	1.0–1.2	Essential micronutrients (A, C, D, E, selenium, zinc), balanced macronutrients, adequate fluids	[117,126]
Chemotherapy-Induced Nutritional Challenges	25–35	1.2–1.5	Vitamin D, zinc, selenium, glutamine, electrolytes	[133]
Radiotherapy-Induced Mucosal and GI Damage	25–35	1.2–1.5	Glutamine, omega-3 fatty acids, vitamin D, probiotics	[134]
Cancer Cachexia and Muscle Wasting Prevention	30–35 (may increase up to 40 kcal/kg/day in severe catabolic states)	1.5–2.0	EPA (omega-3), high-quality proteins (egg, fish, poultry, dairy, soy), complex carbohydrates, monounsaturated fats	[135]
Management of Anorexia and Reduced Appetite	35–40 from energy-dense foods	1.2–1.5	High-calorie supplements, dairy proteins, nut butters, omega-3s	[136]
Taste Alterations (Dysgeusia)	25–30	1.0–1.2	Zinc, citrus-free flavonoid-rich foods	[137]
Nausea and Vomiting Management	25–30 depending on tolerance	1.0–1.2	Ginger, B-vitamins, hydration, electrolyte solutions	[138]
Diarrhea and GI Toxicity Management	25–30	1.0–1.2	Probiotics, soluble fiber (pectin), zinc, ORS solutions	[139]
Constipation (Often Opioid-Induced)	25–30	1.0–1.2	Insoluble fiber, magnesium, hydration	[140]
Bone Health in Hormone or Steroid Therapy	25–30	1.0–1.2	Vitamin D, calcium, magnesium, vitamin K2	[141]
Immune System Strengthening	25–35	1.2–1.5	Vitamin C, zinc, selenium, probiotics, omega-3 fatty acids	[142]

### 4.3. Functional Foods and Supplements in Treatment Outcomes

Functional foods and dietary supplements are becoming progressively acknowledged as one of the factors that help cancer treatments to be more effective through changes in oxidative stress, inflammation, and immune function [143]. These foods are loaded with bioactive compounds like polyphenols, flavonoids, carotenoids, phytosterols, and probiotics that not only provide nutrition but also have physiological protective effects [144]. Probiotics and prebiotics balance the intestinal microbial flora, promote mucosal immunity, and alleviate therapy-related diarrhea. Omega-3 fatty acids such as EPA and docosahexaenoic acid (DHA) lower systemic inflammation, defend against cachexia, and improve treatment response [145]. Phytochemicals such as curcumin, resveratrol, and green tea polyphenols inhibit tumor-promoting signaling pathways like NF-κB and STAT3, which results in reduced cancer cell proliferation and drug resistance [146]. Moreover, vitamin D and calcium supplements are good for maintaining bone and muscle health, particularly in patients undergoing hormone or corticosteroid therapy, while branched-chain amino acids (BCAAs) support protein synthesis and recovery [147]. Conversely, supplementation must be done very cautiously to avoid possible interactions with chemotherapy drugs, e.g., antioxidants that may interfere with reactive oxygen species-dependent tumor cell killing [148]. Firstly, the incorporation of functional foods into individually planned nutritional schemes not only makes the patient physically more capable of withstanding the treatment but also invigorates the immune system, reduces the inflammation, and facilitates a better recovery and survivorship stage in the distant future [149].

## 5. Diet and Prognosis in Long-Term Cancer Management

Diet is one of the main factors that influences the biology of cancer and its prognosis (recurrence, treatment response, and long-term survival) through interconnected metabolic, inflammatory, hormonal, and immune pathways [150]. The human body after the active therapy is still in a state of metabolic instability and oxidative stress, in which nutritional interventions might be able to alter the cellular signaling from relapse to recovery. On a biological level, the connection between diet and cancer prognosis is mediated through the aforementioned systems of metabolic regulation, immune modulation, redox homeostasis, and gut–microbiota interactions that constitute a feedback network that influences tumor dormancy and systemic resilience [29]. Those patients who persistently consume large amounts of refined sugars, trans fats, and processed meat undergo chronic hyperinsulinemia and have elevated levels of IGF-1, thus activating PI3K/Akt/mTOR and RAS/MAPK pathways. These lead to enhanced tumor cell proliferation, angiogenesis, and inhibition of apoptosis [94,151]. On the other hand, nutrient-dense diets with complex carbohydrates, lean proteins, omega-3 fatty acids, and polyphenolic compounds inhibit the oncogenic cascades and promote mitochondrial stabilization, thus allowing cellular repair and immune surveillance to be increased [152].

Energy balance and macronutrient quality remain the main aspects of the recovery process at the metabolic level. Proper caloric intake (25–35 kcal/kg/day) and good quality protein (1.2–1.5 g/kg/day) are factors that promote lean body mass and prevent cancer-induced cachexia [153]. One of the amino acids, namely leucine together with glutamine, provides the pathways of muscle anabolism and immune cell activation. Omega-3 fatty acids (EPA and DHA) are derived from fish oils, and on the other hand, Myeloperoxidase-inhibiting prostaglandin synthesis activity occurs through COX-2 and inhibits IL-6 and TNF-α, among other pro-inflammatory cytokines, thereby restoring the cycle of tumor signaling driven by inflammation [40]. Antioxidant nutrients also have an indispensable molecular role. Vitamins C and E, selenium, zinc, and polyphenols in plants are all antioxidants and are capable of neutralizing ROS generated not only in the process of cancer progression but also in oxidative stress induced by therapy [154]. Like tumor cells, certain dietary flavonoids such as quercetin, catechins, and curcumin, among others, physically bind with transcription factors like NF-κB and STAT3, thereby blocking gene expression related to inflammation, proliferation, and metastasis [155,156].

**Figure 2:**
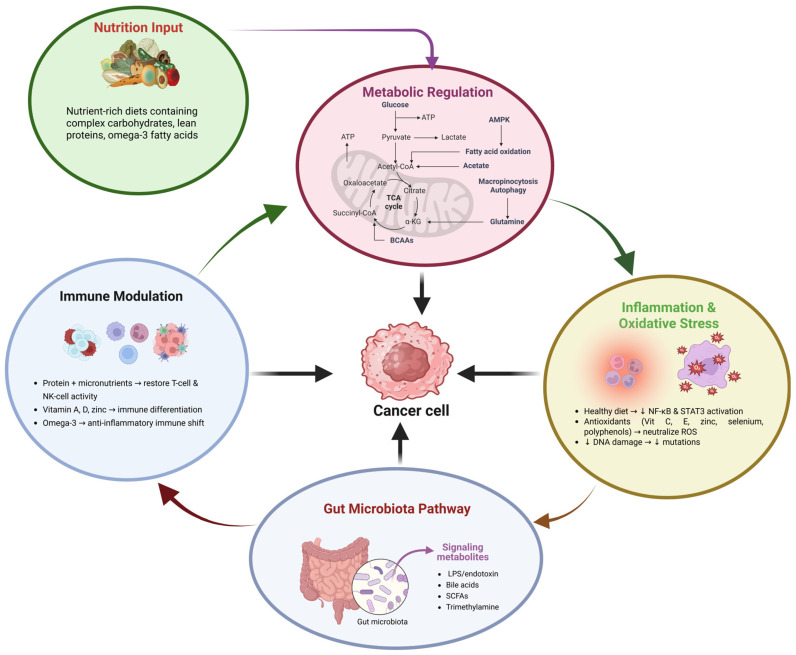
Conceptual model illustrating how nutrition influences cancer biology through interconnected pathways of metabolic regulation, inflammation and oxidative stress, immune modulation, and gut microbiota signaling. Nutrient-rich diets containing complex carbohydrates, lean proteins, and omega-3 fatty acids support metabolic stability and immune function while reducing inflammatory and oxidative damage. These mechanisms collectively influence cancer cell behavior. Colored circles represent major biological pathways: green indicates nutrition input, purple represents metabolic regulation, yellow denotes inflammation and oxidative stress, blue indicates immune modulation, and gray represents gut microbiota signaling. Arrows indicate directional regulatory interactions between these pathways and their combined influence on cancer cells.

The gut microbiome is like another essential biological system that connects diet to the outcome of the disease. The fermentation of dietary fibers by *Bifidobacterium* and *Lactobacillus* leads to the generation of SCFAs, especially butyrate, which are the preferred energy sources for colonocytes and help the epithelial barrier become more resistant, and, at the same time, inhibit HDAC, which therefore leads to tumor suppressor gene expression [157]. A high-fat, low-fiber diet-induced dysbiosis elevates LPS levels, which, after entering circulation, trigger TLR4–NF-κB inflammatory signaling in macrophages and hepatocytes via the interaction with the LPS receptor [158]. Nutrition after treatment also regulates the hormonal and neurobiological pathways that are vital for the patient’s well-being in the long run (Figure 2). The intake of tryptophan, magnesium, and B vitamins in the diet is absolutely necessary for the production of serotonin and dopamine, which in turn relieve the depression and cognitive dysfunction that is frequently referred to as “chemo brain” [159]. Moreover, vitamin D and calcium cooperate in regulating gene transcription by means of the VDR that is responsible for more than 200 genes involved in cell differentiation and apoptosis. Proper hydration and micronutrient supplementation contribute to the maintenance of mitochondrial function and, thus, they are a means of preventing chronic fatigue syndromes that are caused by post-therapy inflammation [160].

## 6. Epigenetic Regulation Through Diet

Epigenetic regulation is a key focus area and among one of the most rapidly changing areas of cancer nutrition research. It is a study concerned with the influence of nutrients and bioactive compounds on gene expression without altering the DNA sequence [161]. The dominant factors determining whether the genes that code for cancer are activated or inactivated are the changes in the cell, such as DNA methylation, histone modification, and non-coding RNA regulation, which in turn have a direct impact on tumor behavior, invasion, and resistance to therapy [162]. In contrast to genetic mutations, epigenetic changes could be reversed, thus making dietary intervention a safe and potentially effective method for modulating cancer risk and treatment response [163]. Dietary cancer nutrition-induced epigenetic changes are associated with physiological processes that govern methylation and acetylation of DNA and histones, thus altering gene accessibility and transcription [164,165]. One-carbon metabolism, which provides methyl groups to DNA methyltransferases (DNMTs), is dependent on several micronutrients such as folate, vitamin B12, vitamin B6, and choline that are the essential components of it [166]. The adequate supply of these nutrients results in the methylation of tumor suppressor genes like p53 and Breast cancer type 1 (BRCA1), which is a way to maintain genomic stability. Conversely, shortages of these nutrients can lead to global hypomethylation, activation of oncogenes, and chromosomal instability [167].

Besides DNA methylation, histone modifications have also been a main focus of cancer epigenetics research. Food nutrients can influence the activities of histone acetyltransferases (HATs) and HDACs by changing chromatin structure [168]. Natural HDAC inhibitors are organic compounds like sulforaphane (from broccoli), butyrate (from gut fermentation of dietary fiber), and epigallocatechin gallate (EGCG) from green tea, which help to promote open chromatin conformation and cancer cells with the reactivation of silenced tumor suppressor genes [168,169]. Along with regulating the SIRT1 (Silent information regulator-1) and p300/CBP pathways, polyphenols such as curcumin and resveratrol also diminish histone acetylation in the promoter regions of pro-inflammatory genes, thereby inactivating cytokines like IL-6 and TNF-α, which are tumor growth-supporting. The interplay of dietary methyl donors with histone modulators uncovers the role of food as a molecular regulator of cancer [168]. MicroRNAs (miRNAs) are small, non-coding RNAs that regulate post-transcriptional gene expression and their abnormal regulation is a major cause of tumor initiation and progression [170]. Nutrition can alter miRNA expression patterns that regulate the cell cycle, apoptosis, and metastasis. For example, resveratrol, quercetin, and genistein elevate the expression of tumor-suppressive miRNAs like miR-34a and miR-200c, which inhibit epithelial–mesenchymal transition (EMT) and metastasis [171,172]. On the other hand, cancer cells may also decrease tumor-suppressive miRNAs and increase oncogenic miRNAs. Furthermore, miR-21 and miR-155 are examples of miRNAs overexpressed in breast, prostate, and colorectal cancers. In addition, omega-3 fatty acids and selenium have been implicated in the regulation of miRNA activity linked to inflammatory signaling and oxidative stress [173,174].

**Figure 3:**
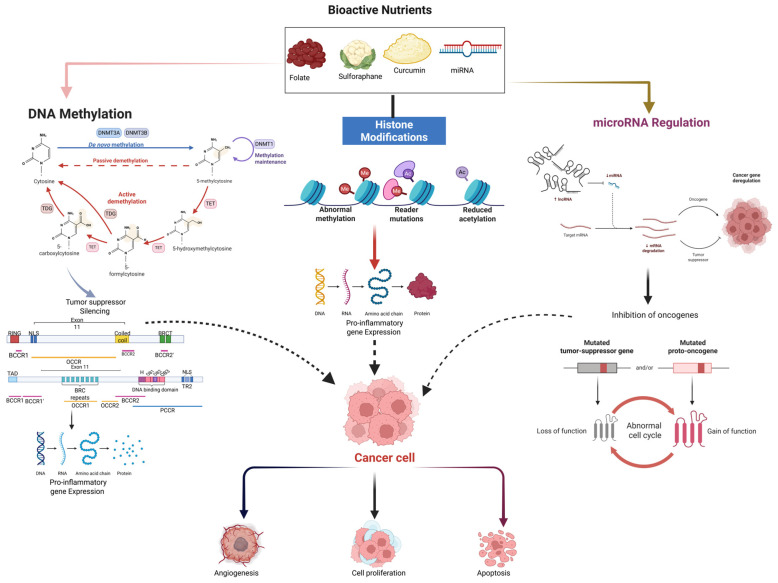
Nutrient-dependent epigenetic changes exemplify the influence of biologically active dietary compounds on DNA methylation, histone modifications, and microRNA regulation, thereby affecting cancer cell behavior. The epigenetic mechanisms depicted here are responsible for changes in the activity of tumor suppressors, the expression of oncogenes, and inflammatory signaling. In general, epigenetic remodeling caused by diet determines angiogenesis, proliferation, and apoptosis in cancer. The colored sections represent the major epigenetic regulatory mechanisms, while arrows indicate the directional interactions and regulatory effects leading to cancer cell outcomes.

One of the main points is that dietary interventions provide a singular chance to change detrimental epigenetic marks. By reprograming epigenetics with nutrition, the cancer cells can be made to heat again as chemotherapy can kill them and the expression of tumor suppressor genes that have been silenced can be brought back [175]. Research has been carried out and the results indicate that the long-term intake of methyl-donor-rich foods, polyphenol supplementation, or short-chain fatty acid-producing diets (that are rich in fiber and probiotics) can have a significant effect on changing methylation profiles and chromatin patterns to the normal ones [176]. These effects do not only turn off the oncogenic transcription but also increase the immune response and metabolic balance. The fields of nutritional epigenomics and metabolite-based chromatin mapping are emerging technologies propagating a move towards real-time gene expression monitoring of dietary influences by researchers [177] (Figure 3).

Besides the independent epigenetic effects of single nutrients, more and more research is pointing to the fact that interactions between nutrients and therapy could play a very large role in cancer-related outcomes [178]. Synergistic interactions between combinations of dietary fiber and probiotics are very evident, where increased production of short-chain fatty acids, especially butyrate, may lead to a stronger acting of histone deacetylase inhibitors and therefore reactivation of tumor suppressor genes [179]. Similarly, polyphenols combined with omega-3 fatty acids may provide anti-inflammatory as well as epigenetic regulatory effects by modulating NF, kB signaling and chromatin remodeling in coordinated pathways. On the other hand, there are also potential antagonistic interactions that must be taken into account. High-dose antioxidant supplementation during chemotherapy may negate the beneficial effect of reactive oxygen species on tumor cell killing, thereby potentially lowering therapeutic efficacy in some situations [180]. Also, too much folate supplementation in the case of established cancer may, in theory, help tumor cells in supporting nucleotide synthesis and thus rapid proliferation. These points stress that context-specific and treatment-aligned nutritional strategies should be prioritized in long-term oncological management [181]. Additionally, excessive folate supplementation in established malignancy may theoretically support nucleotide synthesis in rapidly proliferating tumor cells. These observations highlight the importance of context-specific and treatment-aligned nutritional strategies in chronic cancers [182].

It is widely known that folate, vitamin D, polyphenols, and short-chain fatty acids can epigenetically alter gene expression. However, clinical application is still limited [183]. At present, the clinical practice is to ensure that patients have an adequate micronutrient intake and a high-fiber, plant-based diet which can stimulate the body’s natural production of epigenetically active metabolites such as butyrate [184]. On the other hand, high-dose supplements or specific “epigenetic diets” should not be considered therapeutic interventions outside of clinical trials due to the lack of randomized clinical evidence and potential context-dependent effects [185]. In the coming years, nutrigenomics and metabolomics may personalize dietary intervention of epigenetic pathways, but standardized methods need to be validated through large prospective studies before being incorporated into cancer care guidelines.

## 7. Gut–Brain–Immune Axis and Nutritional Modulation

**Figure 4:**
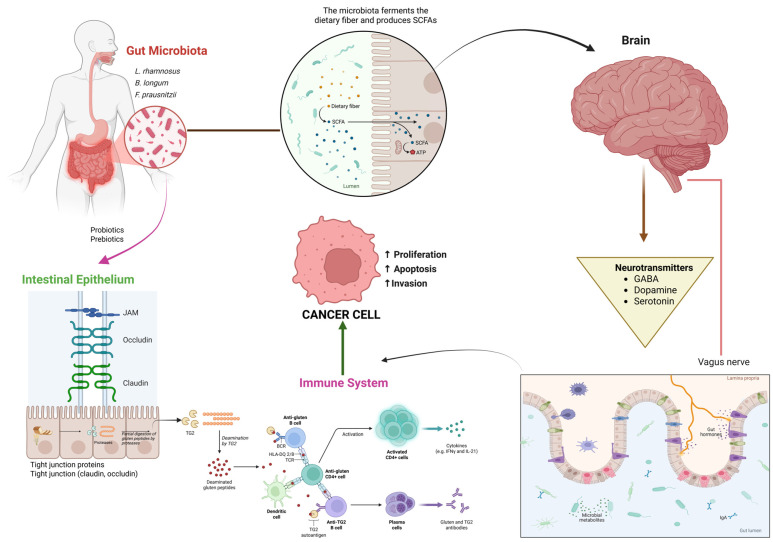
Conceptually, this figure depicts the gut–brain–immune axis as a system where food intake directly influences the gut microbiota composition, the production of SCFA, and the integrity of the epithelial barrier. Immune pathways are adjusted by these microbial signals, which also affect cancer cell proliferation, apoptosis, and invasion. One of the ways with the brain is through neurotransmitters and vagal signaling, thus further regulating systemic inflammation. The interconnected network exemplified here is an integrated network that is nutrition-centered in cancer progression and immune homeostasis. The arrows represent the directional communication and regulatory interactions between gut microbiota, intestinal barrier, immune system, and brain that collectively influence cancer cell behavior.

The gut–brain–immune axis is one of the most intricate and ever-changing communication networks in the human body that involves the intestinal microbiota, central nervous system, and immune function. These are related through biochemical, neural, and endocrine pathways [186]. This axis, in oncological diseases, is instrumental in determining the cancer progression, the response to treatment, and the overall condition of the patient. The gut microbiota pattern affects the body through inflammation, immune tolerance, and neurochemical balance, the main features of which are firmly intertwined with tumor growth, metabolic regulation, and emotional health [187]. As a main factor shaping the composition of the gut microbiota, nutrition is endowed with the capacity to re-establish equilibrium in this axis, to invigorate the immune system, and to effect a combination of better physiological and psychological states not only during the cancer therapy but also in the recovery period [188] (Figure 4).

### 7.1. Microbiota–Immune System Interactions and Metabolic Communication

The gut microbiota is an important immune regulator, affecting not only local mucosal immunity but also systemic inflammatory responses [189]. In cancer, dysbiosis, a condition where there is disturbance of microbial balance, may cause chronic inflammation, immune surveillance that is lessened, and metabolism that is changed [190]. It has been found that the reduction in good bacteria like *Bifidobacterium* and *Lactobacillus*, while at the same time the increase in pro-inflammatory species such as *Fusobacterium nucleatum* and *Clostridium*, can lead to cancer development, particularly in colorectal and gastrointestinal cancers [191,192]. Microbes communicate with immune cells by means of microbial-associated molecular patterns (MAMPs) that bind to TLRs on dendritic cells and macrophages, hence influencing the secretion of cytokines and T-cell differentiation [193]. A healthy diet, rich in fiber, supports microbial diversity and raises the level of SCFAs such as butyrate, acetate, and propionate, which are signaling molecules that help the gut barrier, regulate immune tolerance, and inhibit pro-tumor inflammation [194]. Among other things, butyrate demonstrates anticancer properties, for example, by limiting HDACs; hence, tumor suppressor genes that are silenced are being reactivated and Cluster of differentiation 8 plus (CD8+) T-cell-mediated cytotoxicity is enhanced [195]. These connections form a cycle whereby the diet changes microbial metabolism, microbial metabolites influence immune function, and immune function controls tumor progression [196].

### 7.2. Gut–Brain Communication, Neuroimmune Regulation, and Nutritional Influence

The digestive system is connected to the brain and they send signals to each other via neural, hormonal and biochemical pathways, which together are referred to as the gut–brain axis [188]. This system includes the vagus nerve that conveys microbial and metabolic signals to the central nervous system and neuroendocrine mediators such as serotonin, dopamine, and γ-aminobutyric acid (GABA) that, at least partly, are produced in the gut [197]. Almost 90% of serotonin—a neurotransmitter that stabilizes mood, appetite, and cognition—is produced in the gastrointestinal tract by enterochromaffin cells, and this production is controlled by gut microbes [198]. Food factors have a very deep impact on the neuroimmune balance. Omega-3-rich diets, antioxidative and fermented foods are known to improve vagal tone, increase neurotransmitter production and lower inflammation related to stress condition [199]. On the other hand, a high-fat, low-fiber diet damages the gut wall, increases the amount of LPS in the blood, and causes systemic inflammation that disturbs mood, cognition, and immune defense [200]. The connection between gut bacteria and neurochemistry is essential for cancer patients, where chronic stress and treatment-induced fatigue compromise immune resilience. The brain–immune dialog can be changed by nourishing the gut; thus, depression and anxiety will be alleviated, and treatment tolerance and recovery will be enhanced [201]. Additionally, SCFAs serve as neuromodulators when they penetrate the blood–brain barrier and interact with G-protein-coupled receptors in the brain [202]. They have an effect on microglial activation, oxidative balance, and neuroinflammation through which they indirectly influence tumor–immune interactions. The biochemical crosstalk between the gut and the central nervous system, therefore, is a strong argument for nutritional interventions aimed at the neuroimmune axis in cancer care [203].

### 7.3. Therapeutic Modulation of the Gut–Brain–Immune Axis Through Nutritional Interventions

Diet-driven therapeutic alteration of the gut–brain–immune axis has become a potential consolidated tactic in cancer treatment [204]. By definition, prebiotics, namely inulin-resistant starches, and a variety of oligosaccharides, selectively facilitate the proliferation of beneficial bacteria, which in turn results in greater SCFA production and enhanced immune function [205]. In the same vein, probiotics, *Lactobacillus rhamnosus* and *Bifidobacterium longum*, have been on record as having anti-inflammatory effects, reducing intestinal permeability, and enhancing the patient’s general tolerance to chemotherapy [206]. Microbes are also very instrumental to the achievement of immune checkpoint inhibitors’ goals through the upregulation of antigen presentation and T-cell activation. Psychobiotics—the newest generation of probiotics that primarily focus on mental health issues—control the gut microbiome to alter the mood and stress-eminence skills of cancer patients, thus presenting a double psychological and immunological benefit [207]. So-called “healthy” eating habits such as the Mediterranean diet, being extremely rich in fiber, omega-3 fatty acids, and polyphenols, is capable of supporting microbial diversity, substantially cutting down inflammation throughout the body, and at the same time strengthening both the immune and neuroendocrine systems [208]. The consumption of fermented food substances like kefir, yogurt, kimchi, and kombucha is one sure way to introduce live microbial strains that are capable of working together with dietary fibers to improve gut health [209]. In addition, polyphenols from green tea, turmeric, berries, and grapes are prebiotic-like agents in that they stimulate the growth of SCFA-producing bacteria and, at the same time, regulate oxidative stress in the gut environment [210]. Moreover, studies are gradually showing the effectiveness of microbiome-based therapies such as fecal microbiota transplantation (FMT) and customized microbiome sequencing. Besides dietary intervention, these inventions are ideal measures not only to quiet the gut–brain–immune axis but due to the fact that the gut–brain–immune axis is the way nature connects nutrition to cancer results and diet dysbiosis in cancer patients on immunotherapy [211]. They, together with diet, represent the perfect solutions not only to calm down the gut–brain–immune interface but also to enable a better therapeutic response, less toxicity, and improved quality of life [212]. Being the main agent, besides serving as nourishment, it acts as an active biological regulator of immunity, inflammation, and neurobehavioral health [213].

Mechanistic studies have provided strong evidences for the role of diet in regulating the gut–brain–immune axis, and clinical application is gradually becoming evident [214]. Probiotic and synbiotic supplementation were found to limit the occurrence of chemotherapy-induced diarrhea and other gastrointestinal toxicities in colorectal and breast cancer patients in randomized trials [215]. High-fiber dietary interventions have been linked to better microbial diversity and increased response rates to immune checkpoint inhibitors in immunotherapy through observational studies [216]. Administration of omega-3 has resulted in decreased levels of systemic inflammation and reduced fatigue related to cancer, which points to neuroimmune pathway regulation [217]. Moreover, cases of fecal microbiota transplantation (FMT) combined with immunotherapy have been shown in pilot studies to reinstate sensitivity to treatment in certain patients with refractory disease [218]. Although these results are encouraging, there is still a need for large stratified randomized trials before standardized dietary regimens aimed at the gut–brain–immune axis can be developed to enhance long-term cancer outcomes.

## 8. AI and Digital Innovation in Oncology Nutrition

Artificial intelligence (AI) in combination with digital innovation have been two leading factors that have changed oncology nutrition in a very significant way. The change is basically a shift from the use of traditionally generalized dietary recommendations to precision-based, data-driven interventions that are specifically designed according to the biological profile of the patient [219]. Conventional nutritional assessment methods like manual dietary recalls, food frequency questionnaires, or static blood markers hardly depict the metabolic changes that are happening and the treatment responses of cancer patients [220]. On the other hand, AI-powered technologies are able to achieve this by the use of data analytics, biosensors, and machine learning algorithms which help in generating personalized nutrition strategies that are always up to date with the metabolic state, tumor biology, and the therapeutic progress of a patient [221]. Machine learning models have the ability to analyze large amounts of data including genomic sequences, metabolomic patterns, dietary intake logs, and treatment outcomes to identify complex associations which the clinicians may not be able to notice [222]. One example is that predictive algorithms can identify whether the patient’s tumor is a glycolytic one and thus is likely to respond positively to a low-glycemic or ketogenic diet, while other patients may get the best results from the anti-inflammatory or plant-based regimen [223]. These algorithms make use of reinforcement learning methods to perfect the dietary plans depending on new data collected—they are always adjusting calorie levels, macronutrient ratios, and hydration goals to meet the changing metabolic demands. Besides deeply reducing nutritional precision, the adaptive strategy is also a key factor in lowering the incidences of malnutrition, cachexia, and therapy-related fatigue that are the leading causes of the aggravation of cancer treatment outcomes [219,224].

An additional significant change in cancer medicine created by the fusion of biosensors and wearable technologies is the revolution of personal diet management. The present biosensors are capable of monitoring a variety of health parameters, for example, glucose changes, heart rate variability, body temperature, sleep quality, and even lactate and oxidative stress levels in the body [225]. The AI systems analyze these uninterrupted data streams and react by recognizing metabolic disorders, predicting nutritional deficits, and even suggesting quick intervention solutions. The utility of live data enables nutritionists and oncologists to remotely tailor nutrition plans in such a way that the patients are able to keep up their energy needs and get an optimal intake of nutrients during chemotherapy, radiotherapy, or convalescence [226]. Additionally, the advent of gut microbiota profiling through ultra-sensitive biosensing instruments provides a mass of information regarding microbial diversity, short-chain fatty acids production, and inflammation markers and, hence, makes it easier to establish the connection of diet to immune function and gut health [227]. Another milestone in digital oncology nutrition is AI integration into electronic health record (EHR) systems. By merging nutritional data with lab results, genetic information, and clinical history, AI-powered platforms have the capacity to generate alerts about nutrient–drug interactions or therapy-related metabolic complications on their own [228,229]. In this manner, they can warn about overly high antioxidant intake during chemotherapy, which can interfere with oxidative processes causing cancer cells to die. Moreover, digital twin technology used for prediction modeling, where virtual patient models based on genetic and clinical data are developed, provides researchers with an opportunity to test various diet strategies in silico prior to real clinical trials, thus enhancing precision and reducing risk [230]. Although artificial intelligence is very promising, there are still some issues that need to be solved before it can be widely used in cancer nutrition. One of the biggest problems that needs to be solved are ethical issues, including data privacy, algorithmic bias, and accessibility. In most low- and middle-income countries, the necessary infrastructure for AI-driven dietary management is not available; thus, the gap between the health conditions of different regions of the world may widen even more [231]. Moreover, to keep the nutritionally guided AI systems trustworthy and safe, there is a need for continuous confirmation through clinical trials, and the involvement of oncologists, dietitians, and data scientists working closely together [232].

To reduce inequalities in access to AI-based nutritional tools, it is necessary to have scalable and context-adapted implementation strategies. A mobile-based platform that can work with low bandwidth is just one example, and dietary guidance supported by SMS could be a practical solution for areas with limited digital infrastructure. Public and private partnerships, as well as the integration with community health worker programs, might be among the measures that could help in the decentralized delivery of AI-assisted nutritional screening and counseling. Open-access algorithms based on simple clinical and anthropometric data rather than expensive genomic profiling are likely to make the issue of affordability and adoption easier. Besides that, interfaces in multiple languages and culturally adapted food databases are a must to guarantee that the content is relevant to the different populations. Closing the gap in global oncology care will depend on international collaborations and policy-level investment in digital health equity.

## 9. Emerging Trends and Future Directions

The association between dietary fatty acids and chronic cancer diseases is greatly influenced by considerable inter-individual differences, which should be recognized [233]. Various factors such as the composition of the gut microbiota, genetic variations that affect fatty acid metabolism, exposure to environmental factors, and complex interactions between different foods can alter the biological effects of n, 3 PUFA consumption [234]. Moreover, the majority of epidemiological studies are based on self-reported dietary information, which makes it difficult to accurately measure the real intake level. These aspects make it challenging to directly prove cause and effect, thus indicating the need for research methods that integrate metabolomics, microbiome profiling, and controlled dietary intervention [235]. The future of oncology nutrition is rapidly evolving along three interconnected dimensions: precision, personalization, and integrative care [236]. Precision nutrition would mostly involve adjusting dietary patterns to the tumor biology, genetic variation, and metabolic status of a patient to enhance the treatment effect and alleviate toxicity. Through very precise methods of profiling like glucose monitoring, lipid mapping, and amino acid analysis, medical practitioners are able to figure out the nutrient dependencies that are specific to different types of cancer [237]. Artificial intelligence together with biosensors are now facilitating the on-demand recording of the metabolic responses, hence allowing slow changes in nutrition therapy [238]. For instance, low-glycemic and ketogenic diets have been suggested as efficient ways of cutting off energy supply to glycolytic tumors while omega-3 and polyphenol-rich diets mitigate inflammatory processes and oxidative stress. Nutrigenomics is far beyond what was previously known about the control of nutrients over gene expression, DNA methylation, and tumor suppressor activities through entities like NF-κB and p53 [167]. The metabolomic profiling of tumors is essentially the account of the biochemical features that differentiate the aggressive tumors from the indolent ones; hence, it may serve as a guide for dietary interventions aimed at restoring metabolic equilibrium. Integrative oncology is a healthcare model that embraces nutritional therapy with lactic acid bacteria, fasting-mimicking treatments, and plant-based bioactive substances such as curcumin and resveratrol to improve immune function and treatment tolerance [29]. The studies done so far suggest that regulated fasting may protect normal cells and simultaneously, it may cause tumor cells to become more sensitive to the stress inflicted by chemotherapy. Besides that, diets made according to the gut microbiome may open the way for immune surveillance and anti-tumor activity. Personalized diet regimens combined with the use of machine learning and genomic sequencing are not only becoming adaptable but also anticipatory throughout the different stages of the treatment. Ultimately, the coming of precision nutrition, nutrigenomics, and integrative dietary interventions to oncology is basically the next narrative that speaks of transitioning diet from supportive care to biologically targeted therapy which extends lifespan, prevents relapse, and increases the level of cancer survivors’ quality of life.

## 10. Conclusions

Food has a pivotal and, most significantly, a modifiable influence all along the chronic cancer continuum and, therefore, it impacts cancer risk, tumor biology, treatment tolerance, and long-term survivorship through metabolic, inflammatory, immune, microbiome, and epigenetic pathways. There are indications that plant-based, fiber-rich, antioxidant-dense, and omega-3-enriched dietary patterns are the most consistent in neutralizing carcinogenic signaling while they also provide immune and metabolic resilience. Moreover, a personalized nutrition plan which mainly focuses on energy–protein sufficiency and the intake of functional bioactive compounds during therapy not only makes the patient more capable of tolerating the treatment but also lowers the incidences of complications and alleviates cachexia. After treatment, nutrition remains a factor that determines the risk of cancer coming back through stabilizing redox balance, regulating hormones, and strengthening the gut–brain–immune axis. Rapid developments in nutrigenomics, nutri-epigenetics, microbiome science, and AI-driven digital nutrition are essentially turning diet into a potential co-therapeutic tool rather than a means of supportive care. Nevertheless, the incorporation of these innovations into everyday clinical practice is dependent on stringent trials, the existence of standardized guidelines, and the provision of access to all. In a nutshell, nutrition based on scientific evidence is a low-toxic agent to a patient’s survival, personalization of care, and enhanced quality of life.

**Take-Home Messages**

- Chronic malignancy requires long-term, systemic management integrating metabolic, inflammatory, and immune considerations.
- Dietary patterns rich in fiber, plant bioactives, and omega-3 fatty acids are consistently associated with improved metabolic and inflammatory profiles in cancer contexts.
- Evidence supports nutritional modulation of tumor metabolism and the gut–immune axis, although much remains preclinical or observational.
- Nutrition should be viewed as a low-toxicity adjunct to standard oncology care, not a standalone anticancer therapy.

## Figures and Tables

**Table 1:** Overview of dietary components and their roles in cancer prevention.

Dietary Component	Main Sources	Mechanism of Action	Associated Cancer Effects/Findings	References
Plant-Based Foods and Antioxidants	Fruits, vegetables, legumes, whole grains, nuts, seeds	Rich in antioxidants and phytochemicals that neutralize ROS, protect DNA, regulate detox enzymes, and suppress inflammation	Lower risk of breast, colorectal, and prostate cancers; improved cellular repair and immune response	[53,54]
Dietary Fiber	Whole grains, legumes, vegetables, fruits	Promotes gut microbiota diversity and short-chain fatty acid (butyrate) production; reduces inflammation and enhances immune defense	Decreased risk of colorectal and gastric cancers; improved gut health	[55,56]
Healthy Fats (Omega-3 Fatty Acids)	Fish, flaxseed, chia seeds, walnuts	Anti-inflammatory and anti-proliferative effects; regulate eicosanoid synthesis and suppress angiogenesis	Reduced risk of breast, prostate, and colon cancers; improved metabolic balance	[57,58]
Unhealthy Fats (Saturated and Trans Fats)	Red meat, butter, processed and fried foods	Promote oxidative stress, inflammation, and insulin resistance	Increased risk of colorectal, pancreatic, and endometrial cancers	[59]
Refined Sugars and High-Glycemic Foods	White bread, sugary beverages, sweets	Elevate insulin and IGF-1 levels; stimulate abnormal cell growth and reduce apoptosis	Higher incidence of colorectal, pancreatic, and breast cancers	[60]
Micronutrients (Vitamins and Minerals)	Vitamin A (carrots), C (citrus), D (sunlight/fish), E (nuts), selenium (grains), zinc (legumes)	Support DNA repair, immune modulation, and antioxidant defense	Deficiencies linked with higher risk of breast, colorectal, and prostate cancers	[61]
Bioactive Compounds (Phytochemicals)	Curcumin (turmeric), resveratrol (grapes), EGCG (green tea), lycopene (tomatoes)	Regulate NF-κB, p53, and PI3K/Akt pathways; inhibit inflammation, angiogenesis, and metastasis	Chemopreventive action against multiple cancer types; enhanced apoptosis and reduced tumor growth	[51,62]
Probiotics and Prebiotics	Yogurt, kefir, fermented vegetables, fiber-rich foods	Balance gut microbiota, enhance detoxification, and strengthen mucosal barriers	Reduced colon inflammation and cancer-promoting bacterial metabolites	[63,64,65]

**Table 2:** Tumor molecular features, metabolic dependencies, and potential dietary strategies in long-term oncology.

Tumor Type	Molecular/ Pathological Feature	Metabolic Dependency/ Vulnerability	Potential Dietary Strategy (Mechanism-Linked)	Clinical Evidence Level	Reference
Breast cancer (ER+/PR+)	Hormone receptor-positive tumors often associated with hyperinsulinemia/IGF-1 signaling relevance	Growth signaling via insulin/IGF-1 pathways (PI3K/Akt/mTOR) is a plausible co-driver	Low-glycemic Mediterranean-style diet (reduce postprandial insulin/IGF-1; improve metabolic milieu)	Moderate (human metabolic rationale + trials in progress/biomarker-focused designs; strong epidemiology for glycemic load associations) (PMC (https://pmc.ncbi.nlm.nih.gov/articles/PMC5259892/?utm_source=chatgpt.com, accessed on 5 January 2026))	[80]
Breast cancer (TNBC)	Often more glycolytic and metabolically aggressive; potential chemo-sensitization window	Greater reliance on glucose flux and metabolic plasticity	Low-carb/ketogenic approaches (cautious positioning) as metabolic stressor; omega-3 (EPA/DHA) as adjuvant to modulate metabolism/inflammation and potentially chemo-sensitize	Emerging (clinical evidence limited; mechanistic + early-phase signals; omega-3 supportive evidence stronger than keto) (PMC (https://pmc.ncbi.nlm.nih.gov/articles/PMC10052714/?utm_source=chatgpt.com, accessed on 6 January 2026))	[81]
Breast cancer (HER2+)	HER2 signaling linked to metabolic flexibility and inflammation-related pathways	Lipid signaling + inflammatory mediators may influence tumor biology and therapy response	Omega-3 (EPA/DHA) to shift inflammatory lipid mediators (resolvins; COX-related signaling) and explore subtype-specific biomarkers	Emerging–moderate (biomarker-driven clinical trials exist; clinical endpoints not yet definitive) (prevention.cancer.gov (https://prevention.cancer.gov/clinical-trials/clinical-trials-search/nct02295059?utm_source=chatgpt.com, accessed on 6 January 2026))	[82]
Colorectal cancer (MSI-high vs. MSS)	MSI-high CRC can show glycolysis-linked immune effects; immune microenvironment is central	Tumor-intrinsic glycolysis may contribute to immune evasion/therapy resistance phenotypes	Low-glycemic load/metabolic targeting dietary patterns as adjunct concept (framed as hypothesis-supporting), potentially synergistic with immunotherapy strategies	Emerging (strong molecular evidence; dietary intervention outcomes by MSI status still limited) (PMC (https://pmc.ncbi.nlm.nih.gov/articles/PMC12452990/?utm_source=chatgpt.com, accessed on 6 January 2026))	[83]
CRC (*Fusobacterium nucleatum*–enriched tumors)	*F. nucleatum* enrichment across adenoma–carcinoma sequence; linked with CRC biology and outcomes	Microbiome-driven inflammation and metabolite shifts impacting tumor–immune axis	Fiber-rich + probiotic/synbiotic strategies to reshape microbiota and reduce pro-tumor dysbiosis	Moderate (strong human association + plausible intervention direction; still needs stratified RCTs by F. nucleatum status) (PMC (https://pmc.ncbi.nlm.nih.gov/articles/PMC12525060/?utm_source=chatgpt.com, accessed on 7 January 2026))	[84]
CRC (butyrate sensitivity; stage-context)	Butyrate is an HDAC inhibitor; cancer cells show altered butyrate handling/oxidation	SCFA signaling + epigenetic modulation; altered metabolism in cancerous colonocytes	High-fiber diet/targeted prebiotic–probiotic approaches to increase butyrate production; consider stage/biology nuance in discussion	Moderate (mechanism is strong; human intervention endpoints are still developing) (PMC (https://pmc.ncbi.nlm.nih.gov/articles/PMC6007476/?utm_source=chatgpt.com, accessed on 7 January 2026))	[85]
Prostate cancer (androgen-sensitive/ADT-associated phenotype)	ADT commonly worsens insulin resistance, adiposity, cardiometabolic risk	Systemic metabolic dysfunction can worsen inflammation and overall resilience	Mediterranean/“healthy dietary pattern” + weight management to mitigate ADT metabolic toxicity (support survivorship outcomes)	Moderate (human evidence supports improving metabolic abnormalities in ADT populations) (PMC (https://pmc.ncbi.nlm.nih.gov/articles/PMC9611951/?utm_source=chatgpt.com, accessed on 7 January 2026))	[86]
Prostate cancer (inflammatory microenvironment)	Inflammation and lipid mediators (COX-related) implicated in tumor biology	Eicosanoid balance and inflammation resolution pathways	Omega-3 supplementation (EPA-focused) to shift inflammatory mediators; explore proliferation endpoints pre-surgery	Moderate (clinical) (randomized pre-prostatectomy trial evidence exists for biological endpoints) (Nature (https://www.nature.com/articles/s43856-024-00456-4?utm_source=chatgpt.com, accessed on 8 January 2026))	[87]
Glioblastoma	High glycolytic dependency (classic Warburg-like phenotype); limited metabolic flexibility in many cases	Glucose reliance → potential vulnerability to ketosis-mediated substrate restriction	Ketogenic diet as adjunct (positioned as feasibility/safety + hypothesis for efficacy; avoid overclaiming)	Emerging (phase I feasibility/safety published; efficacy trials still needed) (Nature (https://www.nature.com/articles/s41598-025-06675-6?utm_source=chatgpt.com, accessed on 8 January 2026))	[88]

## Data Availability

No new data were created or analyzed in this study. Data sharing is not applicable to this article.

## Abbreviations

The following abbreviations are used in this manuscript:

WHO	World Health Organization
COX-2	Cyclooxygenase-2 inhibitor
ROS	Reactive oxygen species
IGF-1	Insulin-like growth factor-1
NF-κB	Nuclear factor kappa-light-chain-enhancer of activated B cells
PI3K/Akt/mTOR	Phosphatidylinositol-3-kinase/mechanistic target of rapamycin
EGCG	Epigallocatechin Gallate
RAS/MAPK	Rat sarcoma/Mitogen-activated protein kinases
EPA	Eicosapentaenoic acid
DHA	Docosahexaenoic acid
VDR	Vitamin D Receptor
SOD	Superoxide dismutase
GPx	Glutathione peroxidase
NK	Natural killer
GALT	Gut-associated lymphoid tissue
SCFAs	Short-chain fatty acids
HDAC	Histone deacetylase
LPS	Lipopolysaccharides
PD-L1	Programmed death-ligand 1
IL-6	Interleukin-6
TNF	Tumor necrosis factor alpha
IL-1	Interleukin 1 beta
STAT-3	Signal transducer and activator of transcription 3
BCAAs	Branched-chain amino acids
TLR-4	Toll-like receptor 4
DNMTs	DNA methyltransferases
BRCA-1	Breast cancer type 1
HATs	Histone acetyltransferases
miRNAs	MicroRNAs
CBP	CREB-binding protein
EMT	Epithelial–mesenchymal transition
MAMPs	Microbial-associated molecular patterns
CD8+	Cluster of differentiation 8 plus
GABA	γ-aminobutyric acid
FMT	Fecal microbiota transplantation
AI	Artificial intelligence
EHR	Electronic health record
